# Development of disaster mental health guidelines through the Delphi process in Japan

**DOI:** 10.1186/1752-4458-6-7

**Published:** 2012-07-02

**Authors:** Yuriko Suzuki, Maiko Fukasawa, Satomi Nakajima, Tomomi Narisawa, Yoshiharu Kim

**Affiliations:** 1 Department of Adult Mental Health and National Institute of Mental Health, National Center of Neurology and Psychiatry, 4-1-1 Ogawa-Higashi, Kodaira, Tokyo 187-8553, Japan; 2 National Information Center of Disaster Mental Health, National Institute of Mental Health, National Center of Neurology and Psychiatry, 4-1-1 Ogawa-Higashi, Kodaira, Tokyo, 187-8553, Japan

**Keywords:** Disaster mental health, Delphi process, Guidelines development, Consensus building

## Abstract

**Background:**

The mental health community in Japan had started reviewing the country’s disaster mental health guidelines before the Great East Japan Earthquake, aiming to revise them based on evidence and experience accumulated in the last decade. Given the wealth of experience and knowledge acquired in the field by many Japanese mental health professionals, we decided to develop the guidelines through systematic consensus building and selected the Delphi method.

**Methods:**

After a thorough literature review and focus group interviews, 96 items regarding disaster mental health were included in Delphi Round 1. Of 100 mental health professionals experienced in disaster response who were invited to participate, 97 agreed. The appropriateness of each statement was assessed by the participants using a Likert scale (1: extremely inappropriate, 9: very appropriate) and providing free comments in three rounds. Consensus by experts was defined as an average score of ≥7 for which ≥70% of participants assigned this score, and items reaching consensus were included in the final guidelines.

**Results:**

Overall, of the 96 items (89 initially asked and 7 added items), 77 items were agreed on (46 items in Round 1, and 19 positive and 12 negative agreed on items in Round 2). In Round 2, three statements with which participants agreed most strongly were: 1) A protocol for emergency work structure and information flow should be prepared in normal times; 2) The mental health team should attend regular meetings on health and medicine to exchange information; and 3) Generally, it is recommended not to ask disaster survivors about psychological problems at the initial response but ask about their present worries and physical condition. Three statements with which the participants disagreed most strongly in this round were: 1) Individuals should be encouraged to provide detailed accounts of their experiences; 2) Individuals should be provided with education if they are interested in receiving it; and 3) Bad news should be withheld from distressed individuals for fear of causing more upset.

**Conclusions:**

Most items which achieved agreement in Round 1 were statements described in previous guidelines or publications, or statements regarding the basic attitude of human service providers. The revised guidelines were thus developed based on the collective wisdom drawn from Japanese practitioners’ experience while also considering the similarities and differences from the international standards.

## Background

Japan has a history of large-scale disasters, the most recent of which was the Great East Japan Earthquake that occurred on March 11, 2011. This magnitude 9 earthquake and subsequent tsunami resulted in the loss of nearly 20,000 lives. At the time of this disaster, the Japanese Ministry of Health, Welfare and Labour coordinated the deployment of a disaster mental health team from outside the affected prefectures following requests from local governments there. The team was most active in the early phase of the disaster and has since handed over cases requiring continuous care to local mental health services.

The disaster mental health services were fairly well coordinated by prefectural mental health and welfare centers in terms of assessing the needs in their affected municipalities and requesting dispatch of the disaster mental health team. As is often the case in the time of disaster, however, the workload was overwhelming and there were problems with communications in the affected area. Nevertheless, in our view, the services were coordinated well overall, and this rested on the team’s previous experience of disasters in Japan.

The mental health community in Japan has a wealth of experience in disaster response. In particular, many lessons were learnt from the Hanshin-Awaji earthquake which hit the Kobe area in 1995. Many local government departments of mental health have now prepared a disaster response manual [1], which reference national guidelines published by Kim [2]. The national guidelines were developed and disseminated by the Ministry of Health following a team of experts’ review of the disaster mental health activities conducted following the Hanshin-Awaji earthquake and other natural and man-made disasters. Since their publication, more experience and knowledge has been accumulated following tragic events that occurred in Japan and other countries, such as the Indonesian Sumatra Tsunami and the 9.11 terrorist attacks. In 2007, international guidelines were published after intense discussion by different sectors [3-5]. In light of this and the fact that Japanese mental health professionals have accumulated more knowledge and skills in the decade since Japan’s original guidelines were developed, we sought to develop new guidelines through systematic consensus building and examine the degree of agreement of Japanese experts with the principles of disaster mental health in a systematic manner. In this article, we describe the Delphi process we used to revise the guidelines.

## Methods

(1) Item development: focus group interview

To ensure we had a comprehensive view of mental health and psychosocial care after a disaster, we conducted a thorough literature review. Using PubMed and Google, we searched the scientific literature, guidelines, and manuals, which were written in English or Japanese, using search terms including “disaster”, “emergency”, “mental health”, “psychiatry”, “psychology”, “manual”, and “guidelines”.

To reflect local practitioners’ experience and views, focus group interviews were conducted in three areas which experienced a massive earthquake in Japan, one in an urban area and two in rural areas. Local practitioners with diverse professional backgrounds were invited to attend and represented such professions as psychiatry, psychology, social work, nursing, public health, school counseling, and emergency medicine. Each interview was conducted with 5 to 9 participants (24 participants in total number), with great attention given to the representativeness of the participants as members of mental health teams.

After the three focus group interviews, the contents were transcribed and researchers categorized them into four domains regarding disaster management: 1) the disaster mental health system, 2) initial to early response (from the first week to the first month), 3) management of deployed mental health team, and 4) staffs’ stress management.

(2) Delphi process

The Delphi process is a structured communication technique, originally developed as a systematic, interactive method, which is often used in healthcare fields when scientific evidence is lacking [6]. Participants were recruited from professional networks of the Japanese Society of Traumatic Stress Studies, the Crisis Response Team which is deployed at a time of crisis at schools, and deployed and local mental and community health professionals who have experienced working following massive earthquakes, such as in Kobe, Chuetsu, and Chuetsu-oki in Japan. A total of 100 professionals were invited to join the internet-based survey. Participants invited represented a variety of professionals: clinicians, public health nurses, health authority administrators, and researchers. Many of the local practitioners were themselves survivors of a massive earthquake.

**Figure 1:**
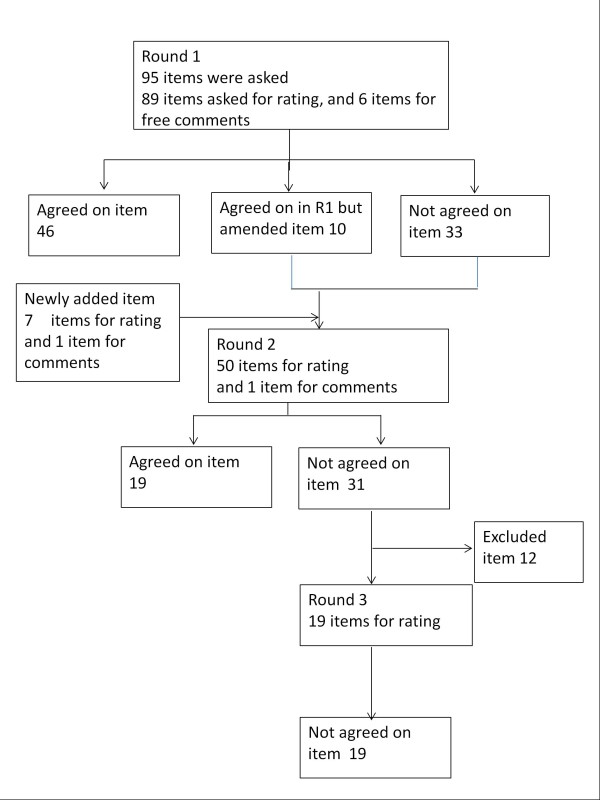
Flow of items asked during the first to third rounds of the Delphi process.

Figure1 summarizes the flow of items asked during the first to third rounds of our Delphi process. In Round 1, our research team provided the participants with an anonymous summary of the items developed from the literature review and focus group interviews, and asked them to rate the appropriateness of the each item on a Likert scale (1: not at all appropriate, 9: very appropriate) and to comment freely on each item. This process was repeated three times via the internet to allow all participants to compare their ratings and comment on others’ ratings.

In Round 2, the survey comprised those statements which did not reach consensus in Round 1. Positive consensus was defined as items for which the mean score was ≥7 and the proportion of participants scoring ≥7 was ≥70%. At this time, participants were provided with summary statistics indicating the number and percentage of participants who rated each score as well as the mean score for each statement. A summary of the comments was inserted underneath each statement for participants to consider when completing the second round. Some statements were amended slightly for clarification, as a result of comments from the first round.

Any statements that achieved positive consensus were removed for Round 3 along with any statements that were unlikely to achieve positive consensus (the proportion of participants scoring ≥7 was <30%). Statements for which the proportion of participants scoring ≥7 was >30% and <70% were retained to determine whether positive consensus could be achieved. These statements were presented in the same format as for Round 2 (i.e. with summary statistics and comments from the previous round). Any items for which the proportion of participants scoring ≥7 was <30% and the mean score was <5 were excluded as an item for which there was no agreement. Participants had access to a list of all participants’ comments and their own scores in Rounds 1 and 2. A full list of the statements is available from the authors on request.

In order to compare the opinions of our Japanese experts with those of European experts, some of the items from The European Network for Traumatic Stress (TENTS) guidelines [7] were included in our Delphi survey. In addition, data on basic characteristics such as sex, age, and professional background were collected from our participants during the online survey.

(3) Analysis

Basic statistics, the proportion of the participants for each item rating, and the mean score of the item rating were generated using STATA 11.0 (StataCorp, College Station, Tx/USA). The comments were summarized by the research team and then the comments and summary were circulated and agreed among the research team members.

(4) Guidelines development

After completing the three Delphi rounds, we drafted the guidelines using all of the statements that achieved consensus. The research team reviewed and clarified each statement when necessary. The draft guidelines were then circulated to the participants for final consensus and any comments. To agree the final wording of the guidelines, the comments were summarized and discussed among the research team and consultants with extensive experience of mental healthcare provision in the aftermath of a natural disaster. The final guidelines are publicly available at http://cocorocare.jp/n/guideline/guideline/.

(5) Ethical consideration

This study protocol was reviewed and approved by the Ethics Committee of the National Center of Neurology and Psychiatry. Upon starting the survey, written informed consent was obtained by letter or email from each participant.

## Results

### Characteristics of participants

From the 100 professionals invited to join the survey, 97 agreed to participate. The response rates were 95.0%, 98.9%, and 93.8% for each round. Psychiatrists accounted for almost one third of participants (n = 28, 29.5%), followed by public health nurses (n = 21, 22.1%), other physicians (n = 13, 13.7%), psychologists (n = 12, 12.6%), psychiatric social workers (n = 11, 11.6%), and other professionals (see Table1). The number of participants slightly decreased as the rounds progressed, but this caused no significant change in the professional backgrounds represented by the continuing participants.

**Table 1:** Characteristics of participants in each Delphi round

	**Round 1**		**Round 2**		**Round 3**	
	**n = 95**	**%**	**n = 94**	**%**	**n = 90**	**%**
Age (years)						
Mean (standard deviation)	48.6	(8.1)	48.7	(8.1)	48.5	(8.2)
Range	28–69		28–69		28–68	
Gender						
Male	48	50.5	46	48.9	44	48.9
Female	47	49.5	48	51.1	46	51.1
Professional background						
Psychiatrist	28	29.5	29	30.9	29	32.2
Other physician	13	13.7	12	12.8	11	12.2
Public health nurse	21	22.1	21	22.3	19	21.1
Nurse	4	4.2	4	4.3	4	4.4
Psychologist	12	12.6	12	12.8	10	11.1
Psychiatric social worker	11	11.6	11	11.7	10	11.1
Administrator	2	2.1	2	2.1	2	2.2
Other	4	4.2	3	3.2	5	5.6

The flow of the three rounds and consensus results is illustrated in Figure1.

#### Round 1

Overall, of 89 items initially asked, 46 (51.7%) achieved consensus and 10 items were agreed on but were amended and further clarification and confirmation asked for. Based on the comments obtained in Round 1, 7 items were newly formulted and asked in Round 2, together with the 33 non-agreed on items.

#### Round 2

In Round 2, 19 items achieved positive agreement and 12 achieved negative agreement (overall agreement rate, 62.0%). Three statements with which participants agreed most strongly were: 1) A protocol for emergency work structure and information flow should be prepared in normal times; 2) A mental health team should attend regular meetings on health and medicine to exchange information; and 3) Generally, it is recommended not to ask disaster survivors about psychological problems at the initial response but ask about their present worries and physical condition. Three statements with which participants disagreed most strongly in this round were: 1) Individuals should be encouraged to provide detailed accounts of their experiences; 2) Individuals should be provided with education if they are interested in receiving it; and 3) Bad news should be withheld from distressed individuals for fear of causing more upset.

#### Round 3

**Figure 2:**
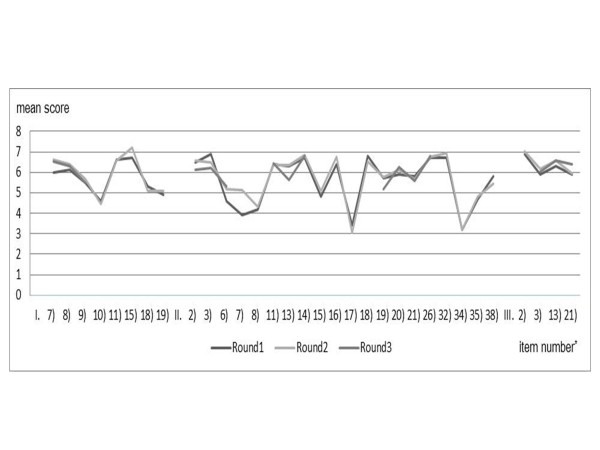
Mean score of the items that did not reach consensus in Rounds 1–3 of the Delphi process.^*^: Statements corresponding to each number are given in Appendix 2.

In Round 3, excluding 12 items for which there was negative agreement in Round 2, 19 items were asked, but none achieved agreement. The changes in mean score of the items that did not achieve conseusus in Rounds 1–3 are shown in Figure2.

## Discussion

### Items that reached consensus

Most of the items that reached consensus in Round 1 were statements which are present in other guidelines or publications, or statements regarding the basic attitudes of human service providers. As experts, our participants answered based on knowledge they had acquired through their own disaster experiences or from workshops, lectures, and publications. Therefore, we consider the statements that reached consensus to be ones that are shared by the wide range of professionals working in disaster mental healthcare. In accordance with the nature of the Delphi process in which consensus is achieved by the majority of participants on specific issues, novel statements did not emerge as agreed-on principles (Appendix 1).

### Items that did not reach consensus

During the survey, more comments were made than initially expected. Such comments included the need to clarify certain statements and specify the assumptions and settings, as well as included practical proposals for specifically defined situations. These comments were made based on the participants’ own experiences, local health structure, and human resource situation, and were therefore extremely valuable. Thus, we decided to include these comments in the final version of disaster mental health guidelines in the form of special articles on controversial items, so that readers of the guidelines could make their own decision on controversial issues.

I. Mental health system during a disaster

First, agreement was not achieved for those items concerning the collection of information needed to set up a disaster mental health system. Specifically, consensus was not achieved for either the item stating that information should be collected first on-site in the disaster area at the expense of a more rapid response or for the item stating that mental health professionals should be deployed to the affected area as soon as possible, even without prior information on the area, to assess local mental health needs. To scout information effectively, a highly experienced team of professionals should be deployed in advance of the entire team to the affected area, and this advance team should become familiarized with the local health resources and services available so they can make best use of them. Alternatively, scouting should be done immediately and jointly involving personnel from both the health and civil sectors.

In Japan’s community healthcare system, in addition to providing primary health care, public health nurses play a major role in providing mental health and psychosocial care [8,9]. In light of this, we asked about several patterns of shared working style for providing mental health services. None of the items reached consensus, and many participants answered that the working pattern used would depend on the circumstances and human resources available, especially in relation to their professional skills and experience of working during a disaster. Some practical suggestions were made in the comments provided: whenever possible, a local and a dispatched public health nurse should work together, although in times of a disaster there is usually a shortage of health staff, which would leave the dispatched nurse to provide outreach to the affected population alone. For effective outreach, it is essential to map cases and resources as well as gather information on the affected area; however, some experts expressed concern about the costs of these necessary preparatory activities for dispatched staff.

Second, the majority of participants expressed reservations about cooperating with the media. The items on the media were adopted from the TENTS guidelines [7] and we found during the Delphi process that some ambiguity remained after the statements were translated into Japanese. Based on comments described, the participants seemed to expect the media to broadcast general information on the disaster and safety issues, broadcast psychoeducational information on people’s reactions (mostly common reactions to an abnormal event) and coping techniques, and some exemplary community recovery efforts. In reality, however, it was noted that the media tends to cover stories that present a biased image of the affected area, journalists tend to be less sensitive to the privacy of the people affected, and mental health personnel feel overburdened by media interviews (usually because they are not well prepared to respond to the media). Practical suggestions for working with the media included establishing an understanding of mental health issues prior to a disaster; for example, special attention should be paid not to cause secondary victimization when interviewing people affected by the disaster, and a representative from the mental health team should be designated to deal with media interviews and other media contact. Thus, it is advised that direct service providers refrain from responding to approaches by the media and instead communicate via a designated team member.

II. Mental health care in times of disaster

In terms of the basic approach to be adopted with disaster survivors, basically the principles of psychological first aid (IASC) were accepted. However, emphasis on promoting sense of efficacy and connectedness was not agreed on. Based on the participants’ comments, the initial response should be focused more on medical needs, with the concepts of efficacy and connectedness in mind. Those who had experience in managing mental health responses to man-made disasters voiced that it was not easy to promote connectedness in a community where there is a perpetrator and victims. It is noteworthy that none of the items stating the role of mental health professionals were agreed on. All of the items were adopted from the TENTS survey [7] and the statements suggest mental health professionals should play a supporting role for professionals at the frontline. Most of the Japanese professionals had firsthand experience in disaster management, thus it is reasonable that they did not agree with a supporting role only. Most of items on initial response did not reach consensus, and 5 of 7 such items were from TENTS guideline. The ambiguous wording of sentences was the main reason for this divergence and a detailed examination of these differences in Japanese and international experts’ opinions will to be described elsewhere.

Statements on screening proved to be another controversial area. Although the importance of screening was endorsed, participants did not reach consensus on how to assess people (e.g., by questionnaire, interview, or formal assessment). Participants suggested that it would depend on the aim of the assessment and the resources and manpower available. Other practical comments recommended that mental health professionals should coordinate a health survey with other actors so that multiple surveys would not be conducted with the affected population. In terms of information provision, the statement that individuals should be provided with education if they were interested in receiving it was not accepted. It was stated that it is not feasible to select affected people with interest in such information and that such selection would carry the risk of stigmatizing person with mental health needs in the community. The idea that volunteers should be recruited and screened for their suitability was not accepted in the survey. Participants expressed concern that there is no valid tool to screen for suitability and that it would be more beneficial to provide orientation on mental health issues for interested volunteers before they begin their activities.

III. Dispatched mental health team

Under the items concerning a dispatched mental health team, discussion centered around who would assume the coordinating role (i.e., at what level, municipal, prefectural, or national). Institutionalization of a coordination mechanism in normal times was seen as key to providing effective services following the Great East Japan Earthquake in 2011. Regarding outreach by the dispatched mental health team, more than 70% of participants agreed that outreach was suitable, but it did not reach the mean score needed to achieve consensus. The reason for not achieving consensus was that many participants agreed with this statement with a rating of ≥7, but a relatively large proportion rated it 5, a neutral position (28.3% for Round 1, 21.7% for Round 2, and 12.4% for Round 3). Agreeing that outreach is an effective approach in the affected area, some participants stated that the dispatched team can only offer temporary help, so local professionals should directly provide outreach to the affected population with support from the dispatched team. Another controversial issue concerned the involvement of mental health professional as volunteers. As professionals who assume clinical responsibility for the care they provide, it is recommended that they work in the affected area within the remits of an organization so that communications involving personal sensitive information can be shared effectively and safely. Other frequent comments were that whether mental health professionals should volunteer depends on the situation, mainly in relation to the availability of such professionals, and on their own clinical skills and discernment.

### Limitations

In accordance with the purpose of the Delphi process, which is transparent consensus generation among experts, generalizability of the sample is not required. However, if participants are selected from an inappropriate sampling frame, biased agreement or lead agreements (i.e., influenced by the surveyor) will likely result. Therefore, careful consideration should be given to the participants recruited and to retain a good participation rate. In this study, we invited experienced professionals from the Japanese Society of Traumatic Stress, the Crisis Response Team, and local and deployed health professionals who had experienced massive earthquakes in Kobe and Niigata, Japan. In terms of their professional backgrounds, the majority worked in medicine, public health, social welfare, or psychology. The quality and quantity of the participants’ disaster response experiences were not strictly controlled for and some of the proposed items were ambiguous and required clarification, and this may reflect the fact that participants understood the item from their own context. In spite of this, these professionals have firsthand experience of responding to disasters and are key members of the disaster mental health team in Japan, and therefore we believe the results obtained in this study represent the realistic views of active team members. However, we acknowledge that involving only service providers can be a weakness. Although some local health professionals voiced their views also as persons affected by disasters themselves, service recipients were not included in our survey. Thus, further exploration on what is needed and what is beneficial should be made from the perspective of service users.

In each Delphi round, a large number of comments was provided and the research team summarized the comments into a 200-word statement for reference in the next round. As there is a risk that the summaries could wrongly lead to and create collective views among the participants [10], the research team members tried to avoid this by creating the summary statements individually and discussing the comments before finalizing the summaries within the team. We included not only the views of the majority of the participants, but also practical suggestions and diverse recommendations received from a few participants in the summaries so that these statements would offer participants the opportunity to rethink the issues in a balanced way, both supportively and critically.

## Conclusion

The response rates were above 90% during the survey and many comments were made in each round, suggesting the participants had good awareness of and were highly motivated to solve the problems the survey addressed. As the participants were deemed to be experts in disaster mental health, this study seems to have successfully gathered their views and prompted some discussion of innovative approaches to disaster mental health. The product of this Delphi process was a new set of guidelines that contains more practical guidance to help mental health workers assigned to work in affected areas problem solve. Guidance is offered in the four domains of 1) the disaster mental health system, 2) initial response, 3) dispatch of a mental health team, and 4) staff care. The newly developed guidelines cover practical issues that were based on the firsthand experiences of Japanese mental health professionals with experience providing mental health care after disasters. Therefore, the new guidelines make a major departure from the previously published ones in that they does not simply conceptualize disaster mental health services, but provide a comprehensive description on what to do and say in times of disaster. We believe that with dissemination and use of the developed guidelines nationally, local mental health systems can be improved and will be better prepared ahead of future disasters.

## Appendix 1 Items that reached consensus

*: Statements asked in the TENTS survey [7].

I. **Mental health system during a disaster**

1. Developing a disaster mental health plan

1) Mental health professionals should be represented on the psychosocial care planning group.*

2) Mental health and community health professionals should have an understanding of the role of and legal rationale for their professional activities as described in related laws and administrative guidelines.

3) Local administration and health and medical facilities should have a business continuity plan and an emergency plan prepared.

2. Setting up a disaster mental health system

4) At the outset of a disaster, local governments should set up a “disaster mental health taskforce”, with local mental health agencies and practitioners implementing the mental health strategy.

5) On setting up a disaster mental health taskforce, it is necessary to have advice from disaster mental health experts or experienced mental health professional to develop a disaster mental health system and action plan.

6) The information system should be centralized at prefectural level to collect disaster-related information and distribute it to affected municipalities.

3. Role of public health nurses.No items achieved agreement.

4. Challenges faced by public health nurses

12) Public health nurses should identify their roles and responsibilities to ensure better liaison with other actors in their routine work in usual times before a disaster occurs (utilization of suicide prevention coordination taskforce, etc.)

5. Documentation of activities and services

14) For a smooth handover among service providers, the disaster mental health activities and services provided should be documented.

16) The psychosocial response provided should be monitored and evaluated by the planning group.*

6. Working with the media

17) To provide appropriate information for media reports, the prefecture should set up an information focal point to manage centralized information output which is tasked with responding to the media.

20) Survivors should not be exposed to or be interviewed excessively by the media. Practical arrangements for this include posting a note asking the media to refrain from accessing survivors without permission.

II. **Mental health care in times of disaster**

7. Basic approach for disaster survivors

1) The initial response should promote a sense of safety.*

4) The initial response should promote calming.*

5) The initial response requires practical, pragmatic support provided in an empathic manner.*

8. Role of mental health professionalsNo items achieved agreement.

9. Initial response

9) Information regarding the situation and concerns of individuals should be obtained and provided in an honest and open manner.

10) Information should be confirmed before responding to queries from the people affected by the disaster, in order not to give them inaccurate or misleading information.

12) Generally, it is recommended not to ask disaster survivors about psychological problems at the initial response but ask about their present worries and physical condition.*

10. Screening

22) Individuals with ongoing mental health difficulties should be offered a formal assessment by a trained practitioner.*

23) Screening for mental health problems should be provided for the assessment of high-risk groups, not for only research purposes.

11. Treatment for high-risk groups

24) Groups at high risk of mental health problems at times of disaster include elderly persons, children, mothers with infants, disabled persons, patients with psychiatric and physical disease, and foreigners.

25) Specialist care is required for specific populations, for example, the elderly and children.*

26) Separate plans are required for specific populations, for example, the elderly and children.*

27) It is desirable to cooperate with the multiple organizations that are providing a response to address the mental health of school-age children, such as schools and school counselors, child consultation offices, and local offices of the Japan Society of Certified Clinical Psychologists.

28) For the mental health care of infants, it is important to respond to parents' anxiety to enable them to bring up children with a stable mind.

29) For the mental health care of infants, it is necessary to provide information about children’s traumatic reactions and care, and provide advice to their parents at evacuation centers, kindergartens, and nursery schools.

12. Information provision

30) A telephone helpline that provides emotional support should be launched.

31) Written leaflets containing education about normal responses to traumatic events and where to seek help if necessary should be provided.*

32) Psychological reactions should be normalised during the initial response.*

13. Staff training

37) All responders should have undergone formal training.*

39) Ongoing supervision of all involved should be provided.*

40) Ongoing training of all involved should be provided.*

41) Different levels of training are required for individuals who are more involved in the psychosocial response.

III. **Dispatched mental health team**

14. Decision on accepting a mental health team from non-affected areas

1) Immediately after a disaster, a mental health team that knows the affected area should be dispatched to severely affected locations for rapid needs analysis.

15. Commencement of the mental health team’s activities

4) The mental health team should attend regular meetings on health and medicine to exchange information.

5) The mental health team should strive to obtain consensus about its policy of care with other actors in health and medicine.

6) A mental health team dispatched from non-affected areas should first obtain information on the disaster and damage before entering the affected area.

16. Activities of dispatched mental health teams

7) A mental health team from non-affected areas should flexibly offer services depending on local needs. (The team should be aware that the members’ own past experiences of disaster response may not be suited to the affected area.)

8) The mental health team should provide services with permission from the local health authority and in collaboration with local mental health resources.

9) A mental health team from non-affected areas should arrange members’ own accommodation, food, and equipment to avoid placing additional burden on the affected area.

10) The mental health team should each comprise a psychiatrist, nurse, public health nurse, psychiatric social workers, and an administrator who takes care of logistics.

11) The mental health team should not readily prescribe medication to the affected population. They should be prescribed by other agencies.

12) A mental health team from non-affected areas should enter the affected area assuming there is no need for mental health care among the affected population.

14) The mental health team should strive to liaise on individual cases with local resources, bearing in mind that it can only offer temporary intervention.

15) The mental health team should offer additional support to affected areas that local mental health professionals cannot fully cover (e.g., providing psychoeducation on traumatic stress).

16) A mental health team from non-affected areas should be attentive to the mental well-being of local health professionals.

18. Mental health professionals wishing to work as volunteers

20) Professionals such as physicians, nurses, and psychologists should be dispatched through an organization so that they can share personal clinical information and work as a team.

IV. **Staff's stress management**

19. Institutionalization of staff care in the affected area

1) A protocol for an emergency work structure and information flow should be prepared in normal times.

2) A service policy concerning staff offers of help to the affected people on the way to work should be predetermined.

3) Regarding staff's work management, especially for taking rests, a manual or training program should be developed and implemented at organizational level.

4) Regarding staff's safety, an information center or procedure to be implemented in a disaster should be predetermined in normal times.

20. Rest and relaxation

5) Staff should work by rotation to ensure there is time for rest.

6) A welfare program and time off for recognition of staff’s hard work should be institutionalized.

7) A space for rest and privacy should be secured for staff at offices or shelters.

21. Support for dispatched staff

8) Review meetings or health check-ups involving screenings and interviews should be arranged for dispatched staff.

9) Time for rest and recuperation should be ensured for dispatched staff.

10) The work of dispatched staff as well as staff who remained to cover the work of the dispatched staff should be recognized and acknowledged by the organization as a whole (e.g., making time to produce a mission report to share experiences within the organization).

22. Self-help for staff

11) Awareness and education of staff about self-care should be fully covered in normal times.

## Appendix 2 Items that did not reach consensus in Round 2

*: Statements asked in the TENTS survey [7]

I. **Mental health system during a disaster**

2. Setting up a disaster mental health system

7) To develop a disaster mental health plan, mental health professionals should be deployed to the affected area as soon as possible, even without prior information on the area, to assess local mental health needs.

8) To develop a disaster mental health plan, mental health professionals should be deployed to the affected area after first collecting information on damage in the disaster area, even at the expense of a more rapid response.

3. Role of public health nurses

9) Public health nurses from the affected area should stay at their health centers to assume a command role, such as information gathering, responding to inquiries, and giving instructions to other staff, instead of providing outreach services themselves.

10) Deployed public health nurses should stay at local health centers to gather information and respond to inquiries, so that public health nurses from the affected areas can directly provide outreach services.

11) Deployed public health nurses should assertively provide outreach services in the affected area, gather information on the area and security of residents, and report their activities to their commander at the local health center.

4. Challenges faced by public health nurses

15) Clinical activities in times of disaster should be documented so that the response provided in the early recovery phase can be reflected in subsequent regular clinical contact.

6. Working with the media

18) Those providing an initial response should work closely with the media.*

19) Those providing an initial response should avoid contact with the media.*

II. **Mental health care in times of disaster**

7. Basic approach to working with disaster survivors

2) The initial response should promote a sense of self and community efficacy.*

3) The initial response should promote connectedness.*

8. Role of mental health professionals

6) Initial support should be provided by non mental health professionals.*

7) Mental health professionals have no role in the initial response.*

8) Mental health professionals should provide an advisory and supervisory role but rarely get directly involved in the initial response.*

9. Initial response

11) It had better to ask the open questions such as “How are you feeling?” when support staff talk to survivors.

13) For the disaster survivor who is overwhelmed by fear and anxiety and stunned, it is more preferred to treat in the empathetic way which provide safety such as snuggling up than to verbalize their feelings.*

14) It is recommended to explain for the survivors and their families who express stress reaction about the common psychological reaction among disaster survivors.*

15) Individuals with high levels of distress should be contacted proactively to maintain contact.*

16) Individuals involved should be contacted proactively, irrespective of their symptoms.*

17) Individuals should be encouraged to provide detailed accounts of their experiences.*

18) Individuals should be neither encouraged nor discouraged from giving detailed accounts.*

10. Screening

19) All individuals should be screened for mental health difficulties using a structured questionnaire or interview.*

20) Formal screening should not occur, but helpers should be aware of the importance of identifying individuals with significant difficulties.*

21) Individuals with difficulties should be formally assessed with consideration for their physical, psychological, and social needs before receiving any specific intervention.*

11. Information provision

32) Psychological reactions should be normalised during the initial response.

34) Individuals should be provided with education if they are interested in receiving it.*

35) Bad news should be withheld from distressed individuals for fear of causing more upset.*

12. Staff training

38) Volunteers should be recruited and screened for suitability before being accepted.*

III. **Dispatched mental health team**

13. Decision on accepting a mental health team from non-affected areas

2) Once local needs are known, a mechanism to dispatch a mental health team should be institutionalized.

3) The mechanism to dispatch a mental health team should be established at prefectural and national levels.

14. Activities of dispatched mental health team

13) A mental health team from non-affected areas should provide outreach to the affected population by visiting homes and shelters.

15. Duration of the mental health team’s activities

18) To maximize its performance, the dispatched mental health team should operate for at least five days, to include handover sessions from and to successive teams.

16. Mental health professionals wishing to work as volunteers

21) Professionals such as physicians, nurses, and psychologists should not offer services individually.

## Authors’ contributions

YS and MF had access to all data from the study and complete freedom to direct its analysis and reporting without influence, editorial direction, or censorship from the sponsors. SN and TN made significant contributions to the conception and monitoring of the survey; YK to survey supervision. All authors reviewed and revised the manuscript for intellectual content and have approved the final version of the manuscript.

## Author details

^1^Department of Adult Mental Health and National Institute of Mental Health, National Center of Neurology and Psychiatry, 4-1-1 Ogawa-Higashi, Kodaira, Tokyo 187-8553, Japan. ^2^National Information Center of Disaster Mental Health, National Institute of Mental Health, National Center of Neurology and Psychiatry, 4-1-1 Ogawa-Higashi, Kodaira, Tokyo 187-8553, Japan.
